# Macrophage activation syndrome due to systemic onset juvenile idiopathic arthritis in a case with liver transplantation

**DOI:** 10.1186/1546-0096-12-S1-P223

**Published:** 2014-09-17

**Authors:** Kubra Ozturk, Mehmet Baha Aytac, Zelal Ekinci

**Affiliations:** 1 Department of Pediatrics Rheumatology, Kocaeli Univertsity Faculty of Medicine, Kocaeli, Turkey; 2 Department of Pediatric Nephrology, Kocaeli Univertsity Faculty of Medicine, Kocaeli, Turkey

## Introduction

Macrophage activation syndrome (MAS) is a serious, potentially fatal complication of rheumatic diseases, which is seen most frequently in systemic onset juvenile idiopathic arthritis (soJIA).

## Objectives

This case is reported to expose the difficulties of diagnosis and management of MAS due to soJIA in a patient with liver transplantation.

## Methods

A 9-year-old girl (with a liver transplantation because of biliar hypoplasia from her father at the age of five years) presented with fever, rash and oligoarthiritis. An urgent tube thoracostomy was performed because of severe pleural effusion. She was transferred to the intensive care unit with the diagnosis of sepsis. On admission, the patient’s white blood cells (WBC) 31600/mm^3^, platelets (plt) 40600/mm^3^, C reactive protein (CRP) 264 mg/dl and liver enzymes was normal. Because of persistent fever and pericardial effusion, bone marrow aspiration was performed and revealed hemophagocytosis. Intravenous methylprednisolone (IV) was administered at a dose of 30 mg/kg/day for three days. Fever was reported to subside and her treatment switched to oral methylprednisolone at a dose of 1.5 mg/kg/day. Maculopapular rash and pericardial effusion persisted. At that time the patient’s hemoglobin (Hb) was 9.2 g/dL, WBC 40900/mm^3^, plt 151000/mm^3^, erythrocyte sedimentation rate (ESR) 8 mm/h, CRP 8.26 mg/dl, fibrinogen 3.7 g/l, alanine aminotransferase (ALT) 93 U/l, aspartate aminotransferase (AST) 69 U/l. After four days, resistant high fever relapsed and dexamethasone, etoposide and IVIG added to her treatment. The dose of tacrolimus given for liver transplantation was increased to 3 mg/day.

## Results

When she was transferred to our clinic, physical examination was notable for temperature ranged from 40° C to above and maculopapular rash. Echocardiography revealed severe pericardial effusion. Hb was 6.1 g/dL, WBC 12600/mm^3^, plt 21800/mm^3^, ESR 4 mm/h, CRP 0.68 mg/dl, fibrinogen 1.2 g/l, D-dimer 19.8 ug/ml, ferritin 57798 ng/ml, ALT 152 U/l, AST 387 U/l. Natural killer (NK) cells were found 1.2 % (normal range 5-23%). Tacrolimus level was severely toxic (> 30 ng/ml) so treatment withdrawn. IV methylprednisolone was administered at a dose of 30 mg/kg/day for three days and switched to prednisolon 2 mg/kg/day. After the tacrolimus level decreased to 5 ng/ml, cyclosporine 5 mg/kg was added. During that time Hb was 9.55 g/dL, WBC 5220/mm^3^, plt 21800/mm^3^, ESR 2 mm/h, CRP 0.13 mg/dl, fibrinogen 1.9 g/l, D-dimer mmax. She was developed headache and dizziness. Because suspicion of central nervous system involment, anti thymocyte globulin (ATG) was administered. MRI which was reported as normal, could be performed the next day. ATG therapy continued because of persisting laboratory findings of MAS. In the fifth day of ATG therapy Hb was 9.41 g/dL, WBC 22000/mm^3^, plt 117000/mm^3^, ESR 2 mm/h, CRP 0.36 mg/dl, fibrinogen 1.1 g/l, D-dimer 1.1 ug/ml, ferritin 1381 ng/ml. Anakinra (1 mg/kg/day) was obtained and added to therapy. After -45- days follow up, she was discharged with prednisolon, cyclosporine and anakinra.

## Conclusion

MAS due to soJIA in a liver transplant patient; although diagnosed in the early phase of the disease, revealed difficulties in the management. Treatment with drugs proposed for hemophagocytic lymphohistiocytosis and increased dosage of tacrolimus did not sustained remission. The patient improved with cyclosporine and ATG. It is emphasized that MAS due to soJIA should be treated urgently with appropriate drugs.

## Disclosure of interest

None declared.

